# Zirconia as a Dental Biomaterial

**DOI:** 10.3390/ma8084978

**Published:** 2015-08-04

**Authors:** Alvaro Della Bona, Oscar E. Pecho, Rodrigo Alessandretti

**Affiliations:** Post-graduation Program in Dentistry, Dental School, University of Passo Fundo, Campus I, BR285, Passo Fundo, RS 99052-900, Brazil; E-Mails: opey@correo.ugr.es (O.E.P.), rodrigo.alle@yahoo.com.br (R.A.)

**Keywords:** biomaterials, zirconia, bonding

## Abstract

Ceramics are very important in the science of dental biomaterials. Among all dental ceramics, zirconia is in evidence as a dental biomaterial and it is the material of choice in contemporary restorative dentistry. Zirconia has been applied as structural material for dental bridges, crowns, inserts, and implants, mostly because of its biocompatibility, high fracture toughness, and radiopacity. However, the clinical success of restorative dentistry has to consider the adhesion to different substrates, which has offered a great challenge to dental zirconia research and development. This study characterizes zirconia as a dental biomaterial, presenting the current consensus and challenges to its dental applications.

## 1. Characteristics of Zirconia

The most popular dental ceramic systems are silica-, leucite-, lithium disilicate-, alumina-, and zirconia-based materials. Currently, zirconia-based ceramics are the most studied, challenging researches for different reasons.

Zirconia (zirconium dioxide, ZrO_2_), also named as “ceramic steel”, has optimum properties for dental use: superior toughness, strength, and fatigue resistance, in addition to excellent wear properties and biocompatibility.

Zirconium (Zr) is a very strong metal with similar chemical and physical properties to titanium (Ti). Incidentally, Zr and Ti are two metals commonly used in implant dentistry, mostly because they do not inhibit the bone forming cells (osteoblasts), which are essential for osseointegration [1]. 

Dental zirconia is, most often, a modified yttria (Y_2_O_3_) tetragonal zirconia polycrystal (Y-TZP). Yttria is added to stabilize the crystal structure transformation during firing at an elevated temperature and improve the physical properties of zirconia. Upon heating, the monoclinic phase of zirconia starts transforming to the tetragonal phase at 1187 °C, peaks at 1197 °C, and finishes at 1206 °C. On cooling, the transformation from the tetragonal to the monoclinic phase starts at 1052 °C, peaks at 1048 °C, and finishes at 1020 °C, exhibiting a hysteresis behavior. The zirconia tetragonal-to-monoclinic phase transformation is known to be a martensitic transformation [2]. During this zirconia phase transformation, the unit cell of monoclinic configuration occupies about 4% more volume than the tetragonal configuration, which is a relatively large volume change. This could result in the formation of ceramic cracks if no stabilizing oxides were used. Ceria (CeO_2_), yttria (Y_2_O_3_), alumina (Al_2_O_3_), magnesia (MgO) and calcia (CaO) have been used as stabilizing oxides. So, as the monoclinic phase does not form under normal cooling conditions, the cubic and tetragonal phases are retained, and crack formation, due to phase transformation, is avoided [2]. It is also important to consider that the stabilization of the tetragonal and cubic structures requires different amounts of dopants (stabilizers). The tetragonal phase is stabilized at lower dopant concentrations than the cubic phase. However, another way of stabilizing the tetragonal phase at room temperature is to decrease the crystal size (the critical average grain size is <0.3 μm) [3]. This effect has been attributed to a surface energy difference [2]. Consequently, zirconia-based ceramics used for biomedical purposes typically exist as a metastable tetragonal partially stabilized zirconia (PSZ) at room temperature. Metastable means that trapped energy still exists within the material to drive it back to the monoclinic phase. It turned out that the highly localized stress ahead of a propagating crack is sufficient to trigger zirconia grains to transform in the vicinity of the crack tip. In this case, the 4% volume increase becomes beneficial, essentially squeezing the crack to close and increasing toughness, known as transformation toughening [2].

## 2. Zirconia Structures for Dentistry

Zirconia structures used for dental purposes are fabricated using CAD-CAM (computer-aided design and computer-aided manufacturing) technology in two possible ways. One method mills the fully sintered block of zirconia with no distortion (shrinkage) to the final structure. The disadvantages are the great wear of the grinding tools (burs) and the population of flaws produced during the machining that may lower the mechanical reliability of the structure [4,5]. In the other method, the zirconia structure is milled from a pre-sintered block, reaching its final mechanical properties after sintered, which produces structural shrinkage that can be partly compensated at the designing stage, and the fit of the zirconia restoration will be warranted [6,7]. Both CAD-CAM processes have three main steps: acquisition of digital data, computer processing and designing, and fabrication of the zirconia structure [8]. Most importantly, the CAD-CAM technique has the ability to produce zirconia restorations with sufficient precision for dental use [9,10]. 

Traditionally, zirconia is dull white in color and its opacity can mask the underneath structure. Most dental zirconia systems indicate structural dyeing (coloring) to enhance the esthetic [11]. Currently, full-contoured (anatomical-shaped) monolithic zirconia dental restorations are offered [12,13], which could abbreviate or extinguish the dental laboratory work on zirconia-based restorations. Several studies reported, however, that Y-TZP would lose its stability in wet environment, leading to strength degradation mostly because of the crystallographic transformation from metastable tetragonal phase to monoclinic phase (T-M transformation) and inherent cyclic fatigue from chewing and para-functional habits (e.g., bruxism and clenching) [2,6,7,14,15]. Nevertheless, the influence of low temperature degradation (LTD) on dental zirconia is still in need of further investigation.

Even so, the most popular zirconia-based restorations have a zirconia infrastructure that is porcelain veneered to adequate anatomic contour and esthetic. There are two main ways of veneering zirconia infrastructures: the traditional layering technique and the hot pressing method [2,7]. Both methods require some sort of porcelain to zirconia bonding. Some studies have observed an exchange of certain chemical elements at the porcelain-zirconia interface, which may contribute to bonding [7,16], but whether a true chemical bonding has formed is yet to be verified. Therefore, micro-or nano-mechanical interlocking is regarded as the major mechanism of porcelain zirconia bonding [17]. On this basis, and with some dispute, sandblasting the zirconia surface before porcelain veneering or resin bonding appears to be the most popular method to promote mechanical interlocking and most reports recommend moderate pressure (around 0.4 MPa) and small particle size [18,19]. Other studies suggest that sandblasting induces monoclinic phase transformation, but it can be reversed by the veneering process [7,20,21]. In addition, primers and liners have been suggested to improve wetting and bonding to zirconia [2,19,22,23].

Nonetheless, one of the most important reasons for introducing monolithic zirconia restorations is the significant rate of porcelain fracture from porcelain veneered zirconia-based restorations (6%–25% after three years), which is greater than the fracture rate reported for porcelain fused-to-metal (PFM) systems [2,24,25,26,27,28,29]. This subject challenged many researchers and triggered few review publications on the fracture rate of all-ceramic restorations [24,27,30], indicating that delamination (failure at porcelain-zirconia interface) and chipping (failure within the veneering porcelain) are the most common modes of failure [30,31]. Studies suggested several possible causes for porcelain failure on zirconia-based restorations [7,15,22,32,33,34,35,36]. 

The mismatch in some mechanical and thermal properties such as fracture toughness, flexural strength, coefficient of thermal expansion, and elastic modulus affect the bonding between porcelain and zirconia [7,34,35,36,37,38]. One study emphasized the effect of strength misfit on the development of delamination, showing that the mode of failure changes according to the porcelain strength and suggesting the use of veneering material with a high flexural strength (over 300 MPa) to improve the reliability of zirconia-based restorations [32].

Significant differences on the coefficient of thermal expansion between the zirconia and porcelain influence in the residual stress distribution during the cooling process affecting the reliability of zirconia-based restorations [7,15,22,33,34,35,38,39,40]. The veneering porcelain will experience a change from a viscoelastic state to a solid form when its temperature is reduced and when it passes through the glass transition temperature (*T*_g_ from 480 °C to 610 °C) [2,7,40]. During cooling after sintering, residual stresses might be generated and influence both the strength of porcelain and interfacial integration. Therefore, most manufacturers recommend slow cooling processes [2,38,39]. 

The initiation and propagation of delamination was also reported to be related to the misfit in elastic moduli and fracture toughness of porcelain and zirconia [36]. 

Pressable veneering porcelains were thought to improve bonding between porcelain and zirconia, but studies are controversial on this matter and most of them showed no significant difference between the traditional and pressing methods [41,42]. New veneering methods using CAD-CAM technology seem to improve wetting and bond strength between zirconia and porcelain [43,44]. 

## 3. Resin Bonding to Zirconia 

At first, one could imagine that an all-ceramic restoration would not withstand the intra-oral service. It could be true if the restoration would not be bonded to the tooth structure or remaining restorative materials (e.g*.*, composites and metals), working as an integrated system where diverse stresses, from chewing to para-functional habits (e.g., bruxism), are distributed throughout the system due to appropriate bonding [2,45]. This rationale is supported by the ISO 6872:2010 [46] standard that classifies the ceramics according to the intended clinical use and made the distinction between adhesively and non-adhesively cemented restorations.

Today, glass ionomer (GIC) and resin-based cements are the primary choices for bonding ceramic restorations to the remaining tooth structure. GIC and resin-modified GIC (RMGIC) are often used to cement acid-resistant ceramics, mostly because these cements are very easy to use. However, the most popular and effective cements for all types of ceramic restorations are the resin-based composites, including the systems containing the 10-methacryloyloxydecyl-dihydrogen-phosphate (MDP) monomer [2,45,47].

It has been reported that the clinical success of resin bonding procedures for cementing ceramic restorations and repairing fractured ceramic restorations depends on the quality and durability of the bond. The former depends upon the bonding mechanisms that are controlled in part by the surface treatment that promotes micromechanical and/or chemical bond to the substrate [6,48,49,50,51,52,53,54,55,56,57,58]. The nonreactive surface of zirconia (acid-resistant ceramic), however, presents a consistent issue of poor adhesion, *i.e.*, low bond strength to other substrates [2].

As zirconia is an acid-resistance ceramic, other methods to produce micromechanical retention have been used, including airborne particle abrasion (APA) systems, often called sandblasting, and coarse diamond rotary instruments. Several studies [17,45,47,50,51,58,59] reported that airborne particle abrasion methods using alumina particles or silica-modified alumina particles (silica coating) produced greater surface roughness (*R*_a_) values and that silica coated surfaces showed a significant increase (76%) in the concentration of silicon, which should enhance bonding to resin via silane coupling agents [45,51,59]. Therefore, silica coating (silicatization) systems (e.g., Rocatec and Cojet, 3M-ESPE) have been used to create a silica layer on metal and ceramic surfaces through high-speed surface impact of the silica-modified alumina particles that can penetrate up to 15 μm into ceramic and metal substrates. This tribochemical effect may be explained by two bonding mechanisms: (1) the creation of a topographic pattern via airborne particle abrasion allowing for micromechanical bonding to resin; and (2) the promotion of a chemical bond between the silica coated ceramic surface and the resin-based material, via a silane coupling agent [2,45].

Therefore, the adhesion between dental ceramics and resin-based composites is the result of a physico-chemical interaction across the interface between the resin (adhesive) and the ceramic (substrate). The physical contribution to the adhesion process is dependent on the surface treatment and topography of the substrate and can be characterized by its surface energy. Alteration of the surface topography results in changes on the surface area and on the wettability of the substrate, which are related to the surface energy and the adhesive potential. In addition, the surface energy of a solid surface is greater than that of its interior where the interatomic distances are equal, and the energy is minimal. In fact, at the surface of the lattice, the energy is greater because the outermost atoms are not equally attracted in all directions. This increase in energy per unit area of surface (J/m^2^ or N/m) is referred to as the surface energy (γ), or surface tension for liquids (e.g., water: γ = 73 mJ/m^2^; PTFE- polytetrafluoroethylene: γ = 18 mJ/m^2^; steel: γ = 230 mJ/m^2^; liquid resin: γ = 40 mJ/m^2^) [2,45]. Therefore, the surface atoms of a solid tend to form bonds to other atoms in close proximity to the surface, reducing the surface energy of the solid. Achieving an energy balance or the lowest energy state is the driving force for the chemical bond between the adhesive and the adherend. However, the surface energy and the adhesive qualities of a given solid can be reduced by any surface impurity or contaminant, such as human secretions and air voids. The functional chemical groups available or the type of crystal plane of a space lattice present at the surface also affect the surface energy [2,45].

Nevertheless, a clean (no contaminants) and dry surface ensures that the adhesive has the best possible chance of creating a proper bond with the adherend. In addition, the wettability of the adherend by the adhesive, the viscosity of the adhesive, and the morphology of adherend surface influence the ability of the adhesive to make intimate contact with the adherend. Thus, to succeed the challenge of resin-bonding to zirconia-based ceramics, one must consider all aspects listed above [2,45]. This rationale has been followed, somehow, in the reports on bonding to zirconia (Table 1). Most experimental procedures that resulted in high bond strength values and had clinical feasibility were tried *in vivo*. Clinical trials on veneered zirconia-based restorations have showed survival rates of 75%–100% [28,29,60,61], which are similar to other successful restorative procedures.

**Table 1:** Experimental studies, in chronological order, on resin bonded dental zirconia.

Study	Dental Zirconia	Aging (Y, yes; N, no)	Test Method	Suggested Bonding Treatment	Mean Bond Strength (MPa)
Janda *et al.*, 2003 [62]	Frialit (Degussit)	Y	shear	Flame-treated for 5 s/cm^2^ (PyrosilPen) + silane	16 ± 6
Blatz *et al.*, 2004 [63]	Procera AllZirkon (Nobel Biocare)	Y	shear	APA + adhesive with MDP + Panavia F	16.8 ± 3.7
Piwowarczyk *et al.*, 2005 [64]	Lava (3M-Espe)	Y	shear	Silicatization (Rocatec)	19.9 ± 2.6
Derand *et al.*, 2005 [23]	Procera Zirkon (Nobel Biocare)	N	shear	Low fusing porcelain pearls + silane	18.4 ± 3.6
Atsu *et al.*, 2006 [65]	Cercon (Dentsply)	N	shear	APA + silicatization (Cojet) + adhesive with MDP + Panavia F	22.9 ± 3.1
Lüthy *et al.*, 2006 [66]	Cercon (Dentsply)	Y	shear	Silicatization (Rocatec) + Panavia 21	73.8 ± 8.5
Kumbuloglu *et al.*, 2006 [67]	DCS (Dental AG)	Y	shear	APA + Silicatization (Rocatec) + Panavia F	20.9 ± 4.6
Blatz *et al.*, 2007 [68]	Lava (3M-Espe)	Y	shear	Silicatization (Rocatec) + Panavia F	16.6 ± 3.2
Wolfart *et al.*, 2007 [69]	Cercon (Dentsply)	Y	tensile	APA + Panavia F	39.2
Aboushelib *et al.*, 2007 [70]	Cercon (Dentsply)	N	microtensile	APA + infiltration etching + Panavia F2.0	49.8 ± 2.7
Yang *et al.*, 2008 [71]	Cercon (Dentsply)	Y	tensile	APA + Panavia F2.0	29.6 ± 4.8
Aboushelib *et al.*, 2008 [72]	Procera Zirconia (Nobel Biocare)	N	microtensile	infiltration etching + silane primer + Panavia F2.0	40.6 ± 5.8
Piascik *et al.*, 2009 [73]	e.max ZirCad (Ivoclar)	N	microtensile	APA + Sílica seed layer + silane + C&B	23.2 ± 5.4
Cavalcanti *et al.*, 2009 [74]	Cercon (Dentsply)	N	microshear	APA + metal primer + BisGMA resin cement	27.9 ± 4.5
Heikkinen *et al.*, 2009 [75]	Procera Zirconia (Nobel Biocare)	Y	shear	Silicatization (Rocatec) + silane + resin cement	4.7 ± 2.7
Aboushelib *et al.*, 2009 [76]	Procera Zirconia (Nobel Biocare)	N	microtensile	infiltration etching + silane primer + Panavia F2.0	41.0 ± 5.8
Oyagüe *et al.*, 2009 [77]	Cercon (Dentsply)	N	microtensile	Silicatization + resin cement with MDP	15.3 ± 3.3
Kitayama *et al.*, 2010 [78]	Cercon (Dentsply)	N	tensile	APA + AZ primer + Resicem	22.3 ± 4.6
Magne *et al.*, 2010 [79]	Lava (3M-Espe)	N	shear	APA + primer + Duo-Link	26.6 ± 6.2
Qeblawi *et al.*, 2010 [80]	e.max ZirCad (Ivoclar)	Y	shear	silicatization + silane + Multilink	30.9 ± 4.6
Jevnikar *et al.*, 2010 [81]	TZ-3YB-E Zirconia (Tosoh)	Y	shear	APA + alumina coating + resin cement	27.3 ± 3.9
Attia *et al.*, 2011 [82]	e.max ZirCad (Ivoclar)	Y	tensile	Silicatization (Rocatec) + silane + Multilink	39.7 ± 7.0
Matinlinna and Lassila, 2011 [83]	Procera All Zircon (Nobel Biocare)	Y	shear	Silicatization (Rocatec) + silane + resin cement	11.7 ± 2.3
Dias de Souza *et al.*, 2011 [84]	Lava Frame (3M-Espe)	N	microtensile	APA + adhesive with MDP + resin cement	6.1 ± 5.3
de Castro *et al.*, 2012 [85]	In-Ceram YZ (Vita)	Y	microtensile	Silicatization (Cojet) + silane + resin cement	13.9 ± 6.0
Lung *et al.*, 2012 [86]	Lava (3M-Espe)	Y	shear	Silicatization (Rocatec) + silane + resin cement	14.5 ± 2.2
Piascik *et al.*, 2012 [87]	Lava (3M-Espe)	N	shear	Plasma fluorination + resin cement	37.3 ± 4.6
Chen *et al.*, 2013 [88]	Cercon (Dentsply)	N	shear	APA + primer with MDP + resin cement	29.0 ± 6.3
Karimipour-Saryazdi *et al.*, 2014 [89]	TZP BIO-HIP (Metoxit AG)	Y	tensile	APA + resin cement	3.7 ± 1.0
Bavbek *et al.*, 2014 [90]	BruxZir and Prettau-Zirkon	N	microshear	Silicatization (Cojet) + silane + resin cement	45.9 ± 4.8
da Silva *et al.*, 2014 [91]	Lava Frame (3M-Espe)	N	microshear	Silicatization (Cojet) + silane + resin cement	37.4 ± 2.3
Oba *et al.*, 2014 [92]	YPS (Kuraray Noritake)	Y	shear	Silane with MDP (Monobond Plus) + resin cement	7.7 ± 2.9
Pereira *et al.*, 2015 [93]	Lava (3M-Espe)	N	shear	APA + primer with MDP + resin cement	14.1 ± 6.1
Şanlı *et al.*, 2015 [94]	In-Ceram YZ (Vita)	N	flexural	APA + resin cement	50.5 ± 1.3
Kim *et al.*, 2015a [95]	KZ-3YF AC (KCM)	N	shear	Zirconia primer + resin cement	10.8 ± 1.5
Abi-Rached *et al.*, 2015 [96]	Lava (3M-Espe)	N	shear	APA + resin cement	7.7 ± 1.1
Oliveira-Ogliari *et al.*, 2015 [97]	Zircon-CAD (Angelus)	N	shear	Silica coating + silane + resin cement	36.7 ± 6.3
Lung *et al.*, 2015 [98]	Upcera (Liaoning)	Y	shear	Silicatization (Rocatec) + silane + resin cement	12.6 ± 2.2
Sciasci *et al.*, 2015 [99]	Lava (3M-Espe)	N	shear	Silicatization (Rocatec) + silane + resin cement	14.9 ± 3.0
Qeblawi *et al.*, 2015 [100]	DiaZir (Wieland)	Y	shear	Silicatization (Cojet) + silane + resin cement	25.6 ± 2.9
Yi *et al.*, 2015 [101]	Lava (3M-Espe)	Y	shear	APA + Z-prime Plus + self-adhesive resin cement	16.5 ± 2.2
Kim *et al.*, 2015b [102]	Cercon Base (Dentsply)	Y	microshear	Adhesive with MDP + resin cement	26.9 ± 6.4
Druck *et al.*, 2015 [103]	In-Ceram YZ (Vita)	Y	shear	Silicatization + silane + resin cement	9.1 ± 4.4

APA-Airborne particle abrasion (sandblasting); water storage was not considered as an aging process.

Considering the *in vitro* studies (Table 1) and the clinical trials, the two most popular clinical strategies to resin bond acid-resistant ceramic restorations are [2,45]:

1. Improving mechanical retention with APA using alumina particles associated to a chemical bonding mechanism using an adhesive/cement system containing ceramic primers, such as phosphate-based monomers, e.g., MDP.
2. Improving mechanical retention with APA using silica-coated alumina particles to introduce an irregular silica layer onto the ceramic surface followed by a silane coupling agent, which promotes a chemical bond to any resin-based adhesive/cement system.

Nevertheless, manufacturers routinely provide cementation recommendations that should be given serious consideration.

However, dental ceramics are, inherently, brittle and can fracture. In general, the most common causes of all-ceramic structural failures are (1) fracture initiated in the connector area of fixed partial dentures (FPDs), either at the core-veneer interface or at the gingival embrasure; and (2) chipping of the porcelain veneer [2,6,24,38,60,104,105,106,107].

The mode of failure is an important aspect of bond strength tests, but it is not commonly reported. A detailed inspection of the fractured surfaces can indicate the failure mode of a bonded assembly. The fracture behavior of adhesive interfaces depends on the stress level, the flaw distribution, material properties, and environmental effects. Therefore, fracture surface characterization combined with analyses of fracture mechanics parameters are of great importance to understand and predict bonded interface reliability and also to reduce the risk for data misinterpretation such as the inference that the bond strength exceed the cohesive strength of the substrate when the fracture initiates away from the interface [2,45,47,51,55]. Therefore, failure analysis based on fractographic principles should assist researchers to correctly interpret the fracture phenomena [2,45,47,55,108,109,110,111], avoiding simplistic comments such as “mixed mode of failure”. Thus, when fractography is correctly used to determine the fracture origin, a proper scientific statement on the mode of fracture can be formulated, improving the quality of the scientific report [2].

The above rationale on adhesion to zirconia should develop the fundamental basis to understand the clinical performance of bonded zirconia-based restorations, the possible failure causes, and the principles to improve the adhesion mechanisms of resin-based composite bonded to zirconia. 
